# Governance, maternal well-being and early childhood caries in 3–5-year-old children

**DOI:** 10.1186/s12903-020-01149-9

**Published:** 2020-06-05

**Authors:** Morenike Oluwatoyin Folayan, Maha El Tantawi, Ana Vukovic, Robert J. Schroth, Micheal Alade, Simin Z. Mohebbi, Ola B. Al-Batayneh, Arheiam Arheiam, Rosa Amalia, Balgis Gaffar, Nneka Kate Onyejaka, Hamideh Daryanavard, Arthur Kemoli, Aída Carolina Medina Díaz, Navneet Grewal, on behalf of the Global Early Childhood Caries Research Group

**Affiliations:** 1 Department of Child Dental Health, Obafemi Awolowo University, Ile-Ife, Osun State Nigeria; 2 Department of Pediatric Dentistry and Dental Public Health, Faculty of Dentistry, Alexandria University, Alexandria, Egypt; 3 Department of Pediatric and Preventive Dentistry School of Dental Medicine, University of Belgrade, Belgrade, Serbia; 4 Department of Preventive Dental Science, Rady Faculty of Health Sciences, Dr. Gerald Niznick College of Dentistry, University of Manitoba, Winnipeg, Manitoba Canada; 5 Department of Child Dental Health, Obafemi Awolowo University Teaching Hospitals Complex, Ile-Ife, Osun State Nigeria; 6 Research Center for Caries Prevention, Dentistry Research Institute, Tehran University of Medical Sciences, Tehran, Iran; 7 Department of Community Oral Health, School of Dentistry, Tehran University of Medical Sciences, Tehran, Iran; 8 Preventive Dentistry Department, Jordan University of Science and Technology, PO Box 3030, Irbid, 22110 Jordan; 9 Department of Community and Preventive Dentistry, Faculty of Dentistry, University of Benghazi, Benghazi, Libya; 10 Preventive and Community Dentistry Department, Faculty of Dentistry, Universitas Gadjah Mada Yogyakarta, Yogyakarta, Indonesia; 11 Department of Preventive Dental Sciences, College of Dentistry, Imam Abdulrahman bin Faisal University, Dammam, Saudi Arabia; 12 Department of Child Dental Health, University of Nigeria, Enugu, Enugu State Nigeria; 13 Dental Service Department, Dubai Health Authority, Dubai, UAE; 14 Department of Paediatric Dentistry and Orthodontics, University of Nairobi, Nairobi, Kenya; 15 Pediatric Dentistry and Orthodontics Department, Universidad Central de Venezuela, Caracas, Venezuela; 16 Amritsar, India

**Keywords:** Preschoolers, Global, Prevalence, Voice and accountability, Control of corruption, Political stability/absence of terrorism

## Abstract

**Background:**

This study assessed the direct, indirect and total effect of distal – political - risk indicators (affecting populations), and proximal risk indicators (affecting women) on the global prevalence of early childhood caries (ECC) in 3–5 year old children.

**Methods:**

Data on global ECC prevalence were obtained from a prior study. Data for distal risk indicators (voice and accountability; political stability/absence of terrorism; control of corruption) were obtained from the World Bank Governance indicators, 2016. Data for proximal risk indicators (women’s opportunity for leadership; percentage of female legislators, top officials and managers; basic employability status of women; ability of women to afford time off work to care for newborns; gross national income (GNI) per capita for females) were derived from the Human Development Index, 2016. Associations between variables were assessed with path analysis.

**Results:**

Voice and accountability (β = − 0.60) and GNI per capita for females (β = − 0.33) were directly associated with a lower ECC prevalence. Political stability/absence of terrorism (β =0.40) and higher percentage of female legislators, senior officials and managers (β = 0.18) were directly associated with a higher ECC prevalence. Control of corruption (β = − 0.23) was indirectly associated with a lower ECC prevalence. Voice and accountability (β = 0.12) was indirectly associated with a higher ECC prevalence. Overall, voice and accountability (β = − 0.49), political stability/absence of terrorism (β = 0.34) and higher female GNI (β = − 0.33) had the greatest effects on ECC prevalence.

**Conclusion:**

Distal risk indicators may have a stronger impact on ECC prevalence than do proximal risk indicators.. Approaches to control ECC may need to include political reforms.

## Background

Early childhood caries (ECC) is one of the predominant health issues among preschool children worldwide. It can have negative consequences on child health, well-being and quality of life [1]. Maternal health and well-being can also affect the child’s oral health [2], and her individual and local community attributes and attitudes can influence the risk for ECC. These attributes include maternal age [3], education, place of employment [4], place of residence [5], employment status [6], and oral health behaviors [7].

Local community, family and individuals’ attributes (proximal factors) are influenced by social, political, and economic factors (distal factors) that impact health and disease outcomes through their influence on the distribution of social resources, such as education, health services, housing, and job opportunities [8]. Additionally, poor health is partly a result of a neglected environment. An environment that does not support maternal well-being impacts negatively on the child’s health [9].

Little is known about how distal factors influence the risk for ECC [10]. A few studies have examined how the interaction between distal and proximal factors affects oral health, but only two focused on ECC [11, 12]. None of these studies assessed how the economic and political environment interact to impact mother’s well-being, which in turn affects a child’s health and risk for ECC.

The theoretical framework for this study is premised on our understanding that external economic and political environments have an impact on women’s literacy, educational attainment and labor market opportunities, and consequently, they affect children’s welfare [13]. Factors such as poverty, ethnic heterogeneity and geographic mobility strongly limit maternal supervision of children [14]. These external factors increase the risk of disadvantaged women’s exposure to poor environments, with more stress, violence, and environmental toxins that negatively influence health [15]. Further, when there is poor representation of women in political spaces, there is low investment in the development of infrastructure that supports maternal health and well-being; resulting in increased child mortality [16].

In a previous publication, we assessed the impact of macro-economic and health care system factors on ECC prevalence [12] and demonstrated how these factors had significant, age-varied impact on ECC prevalence. We are aware that maternal health and well-being affect children’s oral health, but we know little about how the external factors that influence maternal well-being affect ECC risk. Although governance is considered one of the five determinants of health, data on how governance affects the risk for ECC are scarce [17].

We chose to study ECC because of evidence that the oral health profile of preschool children influences their health during childhood and adolescence [18]. The null hypothesis of this study was that the prevalence of ECC in 3–5-year-old children is not associated with factors that affect maternal well-being. Specifically, the study aimed to determine the direct, indirect, and total relationship of distal factors (affecting populations in general), and proximal factors (affecting women’s well-being directly) with the global prevalence of ECC in 3–5-year-old children in the 193 United Nations (UN) member states.

## Methods

Country specific data were collected for the United Nations member states [19] (UN 2017) following the methodological protocol reported by El Tantawi et al. [12]. The proximal and distal structural indicator data were extracted for the latest data between the periods 2007 and 2017. The supplementary file (Additional file 1: Tables 1-8) highlights the details of the distal and proximal structural risk indicators used for this study analysis.

### Data sources

#### Prevalence of ECC in 3–5-year-old children

The data source on the prevalence of ECC was that of El Tantawi et al. [12]. The definition of ECC used was that of the American Academy of Pediatric Dentistry [20]. We analyzed data of 154,452 children aged 36 to71 months from 104 countries. The data were extracted from the Country Oral Health Profile database [21], online database search, and search of master’s and doctoral degree theses about ECC published in local journals and government reports [22]. The search was limited to the period from 2007 to 2017. Data on national prevalence figures were extracted from 25 national surveys. For the countries that had no national surveys, we calculated the ECC prevalence from retrieved data by dividing the number of children affected by ECC in the study(s) by the total number of children examined and multiplied by 100. The data of El Tantawi et al. [11] highlight the details on the prevalence of ECC in the 3–5-year-old children used for this study.

#### Distal risk indicators for maternal health

The 2016 World Bank Governance Index measures the level of governance using six indicators. Three of the six indicators with potential direct or indirect association with ECC were selected (Additional file 1: Tables 1-3). Indicator 1 of the 2016 World Bank Governance Index - voice and accountability - measures perception of how much a country’s citizens participate in selecting their government, have freedom of expression and association, and have free media. Indicator 2 of the 2016 World Bank Governance Index - political stability and no violence/ terrorism - measures perception of the likelihood of political stability and/or absence of politically motivated violence, including terrorism. Indicator 6 of the 2016 World Bank Governance Index - control of corruption - measures perception of the extent to which public power is exercised for private gain. This includes both petty and grand forms of corruption, and “capture” of the state by elites and private interests. The World Bank uses over 30 individual data sources produced from a survey of institutes, think tanks, non-governmental organizations, international organizations, and private sector firms to develop the Index. Countries are given a percentile rank on each of these governance indicators, from 0 (lowest) to 100 (highest). The three indicators all signify positive outcomes: with higher ranks being are indicative of better well-being of the population.

#### Proximal risk indicators for maternal health

These were five factors collected by the United Nations Development Program [23] to report on gender. (1) Women’s opportunity for leadership assessed by the percentage of seats occupied by women in the parliament reported by the 2016 Human Development Index (higher values signify better chances of women in a country to assume leadership positions). (2) Women’s labor market opportunities measured by the percentage of female legislators, top officials and managers of the total in these positions (higher values indicate greater power for women in the job market). (3) Women’s basic employability status measured by the ratio of the percentage of female labor force population aged 15–24 years not in paid employment; or self-employed females available for and actively seeking work, to the percentage of their male counterparts (higher values indicate lower competitiveness of women in the job market). (4) The ability of the system to afford women time off work to care for their newborns, measured by mandatory paid maternity leave in days (countries with higher days of paid leave were considered more supportive of women’s role to care for their children while maintaining their jobs). (5) The level of women’s economic power measured by females’ estimated gross national income per capita (GNI), derived from the ratio of female to male wages, female and male shares of economically active population and GNI (higher value indicated that women had higher income) (Additional file 1: Tables 4-8).

##### Data analysis

The outcome variable was the prevalence of ECC in children 3–5 years old. El Tantawi et al. [12] reported that there are age-distinct risk factors for ECC. We focused on 3–5-year-old children rather than 0–2-year-old children because there were more data for the older age group, thereby increasing the validity of our findings. Statistical analysis included descriptive statistics for the study variables in countries that had data on ECC prevalence and those for which missing data were imputed with stochastic regression. We used stochastic regression imputation to account for uncertainty by using multiple draws from a predictive distribution. In addition to predicting the missing value from a regression equation, stochastic regression calculates a random residual term for each predicted value to preserve variability. Dong and Peng [24] showed that multiple imputation estimated regression coefficients and standard errors for up to 60% missing values. We used per capita GNI to impute missing values of ECC prevalence based on our previous findings of the association between ECC prevalence in 3–5 year old children and GNI [12]. The two data sets (countries with ECC prevalence data and countries with imputed ECC prevalence values) were compared using t-test (Additional file 1: Table 9). The associations between the global prevalence of ECC and proximal and distal risk indicators of ECC were assessed using path analysis.

The path analysis model consisted of three exogenous variables (the three distal risk indicators) and six endogenous variables (the outcome variable and the five proximal risk indicators). We used maximum likelihood estimation and estimated means and intercepts. Prior studies had used path analysis for assessing possible causal relationships using cross sectional data [25, 26]. Bootstrapping was used to generate 95% bias-corrected confidence intervals and standard errors. Standardized estimates (β) were calculated for direct and indirect effects: we calculated these for countries with ECC and for the whole dataset of the United Nations States where missing values were imputed. The model was considered to have a good fit if the chi square test was not statistically significant: the root mean square error of approximation was < 0.05, the comparative fit index was > 0.09 and the Tucker- Lewis index was > 0.09. Significance was set at the 5% level. Statistical analysis was conducted with SPSS version 22.0. The direct links, and direct and total effects were estimated with AMOS version 25.0.

## Results

Table 1 reports the mean values of the proximal and distal risk indicators and prevalence of ECC assessed for the 193 United Nations member states. The mean (SD) prevalence of ECC among 3–5-year-old children was 62.4 (21.4)%. There were no statistically significant differences between countries with prevalence data on ECC and all the United Nations States with imputed values except for countries with ECC data, which had significantly higher GNI per capita for females than did all United Nations countries with imputed values (*P* = 0.008).

**Table 1:** Distribution of proximal and distal factors and ECC in countries with ECC data in 3–5 year olds and UN member states (*n* = 193)

Factor	Mean (SD)		*P* value
	With ECC data	All imputed	
ECC in 3–5 year old children	57.3 (22.4)	62.4 (21.4)	0.07
GNI per capita- females	16,506 (14,735)	11,945 (12,495)	0.008*
Females’ share of parliament seats, % of total	22.3 (11.4)	20.7 (11.5)	0.28
Legislators, senior officials and managers, females % of total	30.3 (9.6)	27.5 (11.0)	0.12
Youth unemployment rate, female: male ratio	1.1 (0.2)	1.1 (0.2)	–
Mandatory paid maternity leave in days	113 (56)	103 (54)	0.17
Voice and accountability, rank	51.8 (29.9)	49.1 (29.1)	0.48
Political stability and absence of violence/ terrorism, rank	45.6 (27.7)	47.5 (28.3)	0.60
Control of corruption, rank	52.7 (30.2)	47.7 (28.8)	0.19

*: Statistically significant at *P* < 0.05

**Fig. 1:**
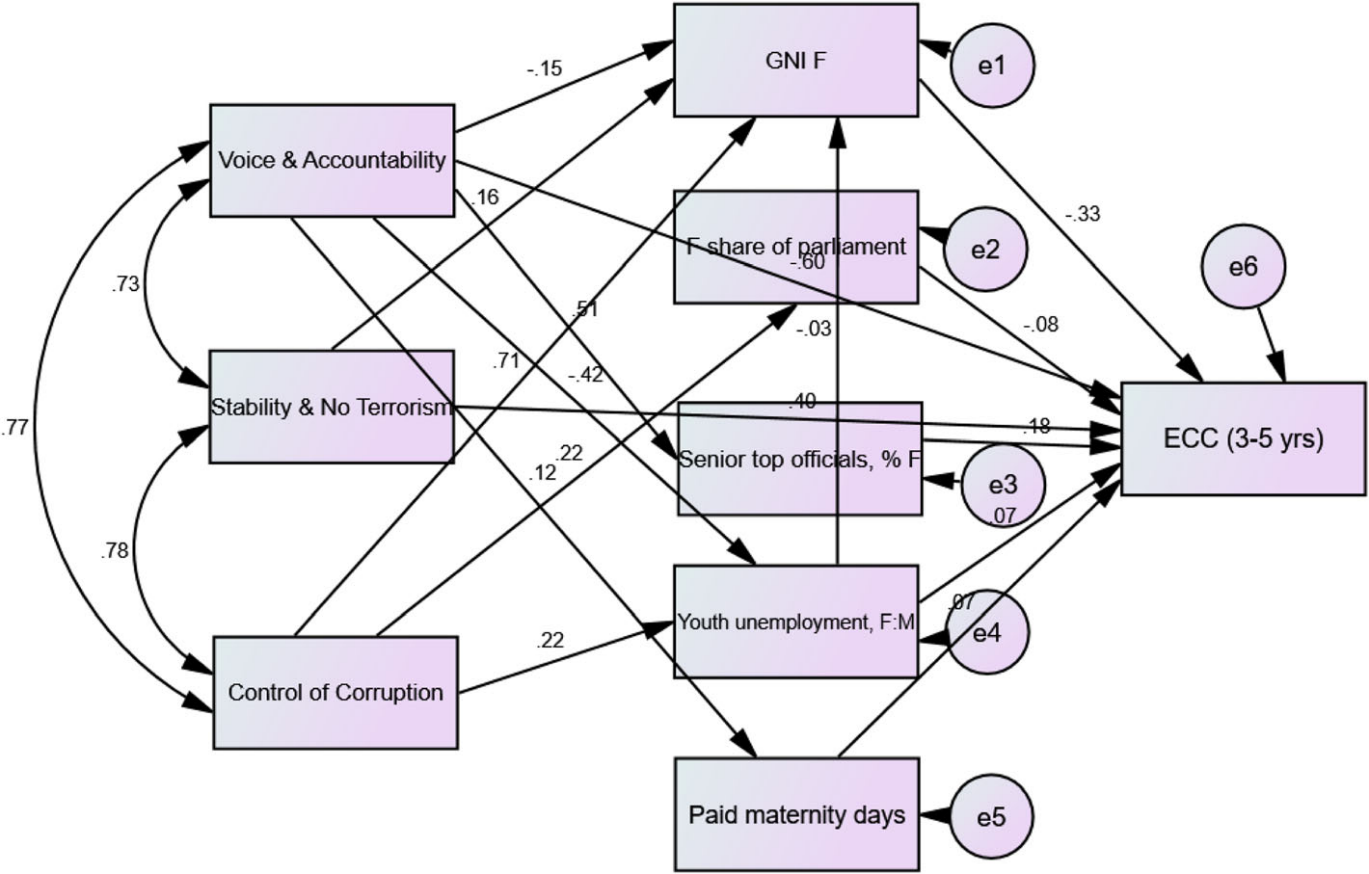
The path analysis model

We developed path analysis models for countries with ECC prevalence data only, and for all countries after imputing the missing values (Table 2). Smaller values of standard errors and narrower confidence intervals in the imputed model indicated greater precision. Figure 1 shows the path analysis model for the imputed model. The Chi square test was 24.30 (*P* = 0.11), the root mean square error of approximation was 0.047, the comparative fit index was 0.989 and the Tucker- Lewis index was 0.977. These results indicated a good model fit. The model accounted for 38.7% of the variation in ECC prevalence in 3–5 year old children.

**Table 2:** The Direct, indirect and total effects of distal factors affecting populations, and proximal factors affecting women on ECC in 3–5 year old children

Variable	With ECC data				All imputed			
	β	SE	P	95% CI	β	SE	P	95% CI
Direct effects								
GNI per capita- females	−0.58	0.18	0.008*	−0.93, − 0.22	−0.33	0.08	0.007*	− 0.47, − 0.14
Females’ share of parliament seats, % of total	− 0.41	0.15	0.04*	− 0.65, − 0.03	− 0.08	0.07	0.24	− 0.23 0.04
Legislators, senior officials & managers, females %	0	0	–	0, 0	0.18	0.07	0.008*	0.04, 0.35
Youth unemployment rate, female: male ratio	0	0	–	0, 0	0.07	0.07	0.32	−0.06, 0.19
Mandatory paid maternity leave in days	−0.28	0.09	0.003*	−0.47, − 0.13	0.07	0.06	0.30	−0.06, 0.19
Voice and accountability	−0.40	0.17	0.03*	−0.73, − 0.07	−0.60	0.09	0.004*	−0.79, − 0.44
Political stability & absence of violence/ terrorism	0.70	0.22	0.007*	0.21, 1.14	0.40	0.11	0.004*	0.19, 0.60
Indirect effects								
Youth unemployment rate, female: male ratio	0	0	–	0, 0	0.009	0.03	0.81	−0.05, 0.06
Voice and accountability	−0.10	0.19	0.79	−0.42, 0.38	0.12	0.06	0.03*	0.006, 0.23
Political stability & absence of violence/ terrorism	−0.21	0.13	0.03*	−0.61, − 0.03	−0.05	0.04	0.09	−0.14, 0.006
Control of corruption	−0.49	0.17	0.02*	−0.84, − 0.17	−0.23	0.07	0.005*	−0.37, − 0.11
Total effects								
GNI per capita- females	−0.58	0.18	0.008*	−0.93, − 0.22	−0.33	0.08	0.007*	−0.47, − 0.15
Females’ share of parliament seats, % of total	−0.41	0.15	0.04*	−0.65, − 0.03	−0.08	0.07	0.24	−0.23, 0.04
Legislators, senior officials & managers, females %	0	0	–	0, 0	0.18	0.07	0.008*	0.04, 0.35
Youth unemployment rate, female: male ratio	0	0	–	0, 0	0.08	0.07	0.28	−0.08, 0.20
Mandatory paid maternity leave in days	−0.28	0.09	0.003*	−0.47, − 0.13	0.07	0.06	0.30	−0.06, 0.19
Voice and accountability	−0.50	0.17	0.01*	−0.80, − 0.15	−0.49	0.10	0.003*	−0.69, − 0.32
Political stability & absence of violence/ terrorism	0.49	0.19	0.02*	0.14, 0.84	0.34	0.10	0.003*	0.14, 0.56
Control of corruption	−0.49	0.17	0.01*	−0.84, − 0.17	−0.23	0.07	0.005*	−0.36, − 0.11

*SE* Bootstrapped SE of β

95% CI: Bias corrected 95% confidence interval

*: statistically significant at *P* < 0.05

The imputed model in Table 2 shows that political indicators had the strongest significant direct association with the prevalence of ECC; higher perception of voice and accountability was associated with lower prevalence of ECC (β = − 0.60). The perception of political stability and absence of terrorism was associated with higher prevalence of ECC (β = 0.40). Other proximal risk indicators had significant effects, but less impact; countries with higher female GNI had lower ECC prevalence (β = − 0.33) and those with higher percentage of female legislators, senior officials and managers had higher prevalence of ECC (β = 0.18).

Control of corruption, had the strongest significant indirect association with the prevalence of ECC as shown in Table 2. A higher perception of controlling corruption was indirectly associated with lower prevalence of ECC (β = − 0.23), whereas voice and accountability was indirectly associated with a higher prevalence (β = 0.12). Political stability/absence of terrorism had an indirect preventive association with the prevalence of ECC although this association was not statistically significant (β = − 0.05, *P* = 0.09). The three political factors directly and strongly affected each other (β = 0.73, 0.77 and 0.78).

Distal (political) factors [voice and accountability (β = − 0.49), political stability and absence of terrorism (β = 0.34) and control of corruption (β = − 0.23)] had a stronger association with the prevalence of ECC than did proximal factors [female GNI (β = − 0.33), percentage of female legislators, senior officials and managers (β = 0.18)].

## Discussion

We examined the direct and indirect association of governance risk indicators of maternal wellbeing on the prevalence of ECC in 3–5-year-old children. This is the first study to provide suggestive evidence of a direct effect of governance-related factors on the prevalence of ECC, and the possible indirect effect of these risk indicators on the prevalence of ECC through its impact on proximal factors that affect women’s health and wellbeing. Our results showed that distal risk indicators may have a greater impact on the prevalence of ECC than do proximal risk indicators affecting women’s health and well-being. Also, we observed that a single risk indicator could have both direct and indirect impact on ECC prevalence, with these effects occurring in opposite directions. The findings support the rejection of the null hypothesis of the study.

Our findings provide insight into the possible impact of respect for human rights on oral health. The results suggest that respect for human rights, reflected in higher perception of voice and accountability (greater freedom of expression, freedom of association and a free media), are associated with lower global prevalence of ECC. This association aligns with evolving evidence that respect for human rights has a positive impact on health [27] through the support of social movements that aim at changing the systems that control health outcomes [28]. When these movements are welcome and respected, new structures evolve, some of which may reduce the prevalence of ECC. Conventionally, the public health response model promotes a ‘welfaristic’ approach to health management [29]. The findings of this study suggest that (dis) respect for human rights has an impact on oral health outcomes, and should be included in the health response models. There are limited studies on how human rights indicators and public oral health indicators interact to affect oral health. Understanding these interactions might promote global health responses that advocate for democratic governances [30] and respect for human rights.

Corruption undermines respect for human rights and affects children’s health [31]. In the present study, the perception of control of corruption was indirectly associated with lower prevalence of ECC. Corruption also undermines the ability of countries and institutions to improve women’s wealth, a factor that we found in this study, which has a direct protective effect on the prevalence of ECC. Prior studies have shown a protective effect of household wealth on oral health [32] and children’s oral health quality of life [33]. Improved maternal wealth may increase children’s access to preventive and curative dental health care, thereby reducing the risk of ECC [34]. These findings suggest that the economic empowerment of women is key to achieving fair distribution of oral health in children, which corroborates our finding that control of corruption has a strong direct effect on female GNI.

Paradoxically, while a higher perception of voice and accountability had a direct protective effect against ECC, it had a minor indirect promoting effect on ECC. This paradox might be attributed to the indirect effect mediated by the percentage of female legislators, senior officials and managers; countries with higher voice and accountability are likely to have higher percentage of female legislators, senior officials and managers. The percentage of women in these positions had the strongest direct and promoting effect on the prevalence of ECC among proximal factors. Since the presence of more women in senior management positions fosters economic growth and increases political stability [35], it is difficult to explain how a higher percentage of women in senior management would be directly associated with higher prevalence of ECC. One possibility is that more women occupying senior management positions reside in politically affluent nations where the risk of ECC is higher [12, 36]. Also, with higher percentage of women in the work force, women who are the primary care providers for children, may have less time to spend with their children, with negative impact on child’s health [37]. It also may imply that in order to reduce the global prevalence of ECC, governments need to support women striving to improve their earnings through establishing work conditions that enable them to afford the time to supervise their children and at the same time compete in the labor market. Efforts to improve women’s political participation may also imply that support for child oral health care is equally important. Also, the oral health care competencies of institutional daily childcare service providers needs to be improved so they can play improved supportive roles for women in the work force. For this reason, the training and competency certification of childcare providers needs to include ability to institute preventive oral care for pre-school children.

Political stability/absence of violence was directly associated with higher prevalence of ECC. This association does not imply that political violence/terrorism prevents ECC. Rather, the association may be attributed to the fact that political stability increases economic growth [38] with manifestations of affluence, such as access to free sugar [39], which increases the risk of ECC. There is evidence that sugar availability decreases during war times [40] and although sugar is more commonly found in politically stable countries, the poorer segments of the population are more likely to be affected by the consumption of free sugar [36]. It would be useful to conduct studies on how political stability, governance, and corruption interact with the commercial determinants of oral health, and how the interactions affect the availability of sugars.

We found that distal risk indicators had a larger impact on the prevalence ECC than did proximal risk indicators. This difference implies that improvement in country governance may reduce the prevalence of ECC more than simply addressing more proximal individual factors. While our results reinforce the finding of Hu and Mendoza [41], who highlighted that governance has a direct impact on child health, it also provides new evidence suggesting that governance had direct impact on child oral health. Like Hu and Mendoza [41], we emphasize that good governance may improve public health funding and thus, improve children’s oral health. We demonstrated that good governance is a composite of actions that independently influence child oral health in different ways and with different magnitude. The collective effect is however, positive.

These study findings indicate that it may be important to develop specific micro- and macro-level indicators to monitor the effects of social and political factors on the oral health of both mother and child. These monitoring indicators will generate evidence on how governance can affect maternal and child oral health and wellbeing.

This study is not without limitations. First, the presence of some confounders might have warranted adjustment because of possible association with the prevalence of ECC. The study had limited ability to make such adjustments however, as the evidence of the impact of macro-level factors on the prevalence of ECC is just evolving, with limited understanding of which macro-level factors affect the prevalence of ECC. Second, ECC prevalence data were missing for some countries; we addressed this deficit by imputing the missing values. This imputation is based on hypothetical modelling analytical technique. We compared the path analysis models with and without imputation, and the models gave similar results with the imputation-based model having more realistic estimates and measures of precision, indicating that it is liable to less bias. Third, although we used a path analysis for assessing possible causal relationships, this analysis was a cross sectional study: cross-section studies have limitations for determination of causal relationships. Future longitudinal studies should be able to generate data for constructing a more accurate causal model. Fourth, as noted by El Tantawi et al. [12] the included studies in the determination of country-level ECC prevalence were those that followed the American Academy of Pediatric Dentistry’s definition for ECC [20], thereby minimizing variation attributable to different diagnostic criteria; however, the possibility of variation in representativeness of the data cannot be ruled out. A similar approach for generating national data was used by the World Health Organization [21]. Fifth, our use of the measure ‘mandatory paid maternity leave in days’ have reflected more effect on the oral health of 0–2-year-old children than on the oral health of 3–5--old children. This may explain why this factor was not found a significant risk indicator for ECC in the studied age group. This problem reflects the limitation of using proxy measures as is often the case with ecological studies, thereby increasing the risk for ecological fallacies.

## Conclusion

This study provides new evidence that the control of ECC may benefit from actions taken at the national level to improve the wellbeing of populations in general and women in particular, which in turn, could improve children’s oral health. We demonstrated that broader political circumstances that serve as causes-of-cause help policy makers and planners appreciate the limitation of individual choice, life-course, and intrinsic cultural-behavioral factors as determinants of risks for ECC. Therefore, for ECC to be controlled on a global scale, actions beyond downstream approaches that focus on individual and family-level interventions need to be undertaken, such as upstream political actions. Oral health stakeholders also need to push for government reforms that might decrease the global prevalence of ECC.

## Supplementary information

**Additional file 1 Tables S1-3:** Factors affecting population. **Tables S4-8:** Factors affecting women. **Table 9:** Comparison between countries with and without missing ECC data.

## Data Availability

Study related materials are public data. All study related data are included in the supplemental file of this manuscript.

## Abbreviations

ECC: Early Childhood Caries
GNI: Gross National Income
