# Supplementary material for: Governance, maternal well-being and early childhood caries in 3–5-year-old children
Source: BMC Oral Health. 2020 Jun 5;20:166. doi: 10.1186/s12903-020-01149-9 (PMC7275475; doi:10.1186/s12903-020-01149-9)
Supplement: Supplementary file 1 — Additional file 1 Tables S1-3: Factors affecting population. Tables S4-8: Factors affecting women. Table 9: Comparison between countries with and without missing ECC data. [file 12903_2020_1149_MOESM1_ESM.docx]

Supplementary data

***Effect of governance on maternal well-being and global prevalence of early childhood caries in 3-5 year olds***

1. Factors affecting population

**Table 1: 1^st^ indicator: Voice and Accountability**

| Country/Territory | Percentile rank | Country/Territory | Percentile rank |
| --- | --- | --- | --- |
| Afghanistan | 21.18 | Libya | 11.82 |
| Albania | 51.72 | Liechtenstein | 87.19 |
| Algeria | 23.65 | Lithuania | 76.35 |
| Andorra | 87.68 | Luxembourg | 96.55 |
| Angola | 16.75 | Macedonia, FYR | 38.42 |
| Antigua and Barbuda | 68.47 | Madagascar | 37.44 |
| Argentina | 65.52 | Malawi | 48.28 |
| Armenia | 30.54 | Malaysia | 33.00 |
| Australia | 94.09 | Maldives | 25.62 |
| Austria | 93.10 | Mali | 39.90 |
| Azerbaijan | 7.39 | Malta | 88.18 |
| Bahamas, The | 74.88 | Marshall Islands | 89.66 |
| Bahrain | 8.37 | Mauritania | 24.63 |
| Bangladesh | 31.03 | Mauritius | 73.89 |
| Barbados | 84.73 | Mexico | 43.84 |
| Belarus | 10.34 | Micronesia, Fed. Sts. | 85.71 |
| Belgium | 95.57 | Moldova | 45.81 |
| Belize | 69.95 | Monaco | 72.91 |
| Benin | 63.05 | Mongolia | 60.10 |
| Bhutan | 44.83 | Montenegro | 49.26 |
| Bolivia | 46.80 | Morocco | 29.06 |
| Bosnia and Herzegovina | 40.89 | Mozambique | 33.99 |
| Botswana | 59.11 | Myanmar | 24.14 |
| Brazil | 61.58 | Namibia | 66.50 |
| Brunei Darussalam | 23.15 | Nauru | 64.04 |
| Bulgaria | 59.61 | Nepal | 38.92 |
| Burkina Faso | 48.77 | Netherlands | 98.52 |
| Burundi | 7.88 | New Zealand | 97.04 |
| Cambodia | 17.73 | Nicaragua | 30.05 |
| Cameroon | 21.67 | Niger | 34.48 |
| Canada | 96.06 | Nigeria | 35.96 |
| Cape Verde | 78.33 | Norway | 100.00 |
| Central African Republic | 18.72 | Oman | 20.20 |
| Chad | 12.32 | Pakistan | 28.57 |
| Chile | 76.85 | Palau | 90.15 |
| Colombia | 49.75 | Panama | 65.02 |
| Comoros | 39.41 | Papua New Guinea | 52.71 |
| Congo, Rep. | 17.24 | Paraguay | 45.32 |
| Congo, Dem. Rep. | 10.84 | Peru | 55.67 |
| Costa Rica | 85.22 | Philippines | 50.74 |
| Côte d'Ivoire | 36.45 | Poland | 72.41 |
| Croatia | 64.53 | Portugal | 86.21 |
| Cuba | 6.40 | Qatar | 15.76 |
| Cyprus | 82.76 | Romania | 63.55 |
| Czech Republic | 80.79 | Russian Federation | 15.27 |
| Denmark | 98.03 | Rwanda | 14.78 |
| Djibouti | 12.81 | St. Kitts and Nevis | 81.77 |
| Dominica | 75.86 | St. Lucia | 83.25 |
| Dominican Republic | 52.22 | St. Vincent and the Grenadines | 80.30 |
| Ecuador | 37.93 | Samoa | 71.43 |
| Timor-Leste | 54.19 | San Marino | 89.66 |
| Egypt, Arab Rep. | 14.29 | São Tomé and Principe | 60.59 |
| El Salvador | 55.17 | Saudi Arabia | 3.94 |
| Equatorial Guinea | 1.97 | Senegal | 57.64 |
| Eritrea | 0.99 | Serbia | 53.20 |
| Estonia | 88.67 | Seychelles | 51.23 |
| Ethiopia | 8.87 | Sierra Leone | 42.36 |
| Fiji | 46.31 | Singapore | 36.95 |
| Finland | 99.01 | Slovak Republic | 75.37 |
| France | 82.27 | Slovenia | 77.34 |
| Gabon | 22.66 | Solomon Islands | 62.56 |
| Gambia, The | 13.79 | Somalia | 2.96 |
| Georgia | 53.69 | South Africa | 67.98 |
| Germany | 94.58 | Spain | 81.28 |
| Ghana | 67.49 | Sri Lanka | 42.86 |
| Greece | 68.97 | Sudan | 3.45 |
| Grenada | 73.40 | South Sudan | 5.42 |
| Guatemala | 34.98 | Suriname | 61.08 |
| Guinea | 26.11 | Swaziland | 9.36 |
| Guinea-Bissau | 27.59 | Sweden | 99.51 |
| Guyana | 56.16 | Switzerland | 97.54 |
| Haiti | 26.60 | Syrian Arab Republic | 1.48 |
| Honduras | 33.50 | Tajikistan | 4.93 |
| Hungary | 57.14 | Tanzania | 40.39 |
| Iceland | 95.07 | Thailand | 20.69 |
| India | 58.62 | Togo | 32.02 |
| Indonesia | 50.25 | Tonga | 69.46 |
| Iran, Islamic Rep. | 11.33 | Trinidad and Tobago | 66.01 |
| Iraq | 22.17 | Tunisia | 56.65 |
| Ireland | 93.60 | Turkey | 29.56 |
| Israel | 71.92 | Turkmenistan | 0.49 |
| Italy | 79.31 | Tuvalu | 83.74 |
| Jamaica | 70.44 | Uganda | 27.09 |
| Japan | 77.83 | Ukraine | 47.29 |
| Jordan | 25.12 | United Arab Emirates | 19.21 |
| Kazakhstan | 13.30 | United Kingdom | 90.64 |
| Kenya | 41.87 | United States | 84.24 |
| Kiribati | 78.82 | Uruguay | 86.70 |
| Korea, Rep. | 67.00 | Uzbekistan | 2.46 |
| Korea, Dem. Rep. | 0.00 | Vanuatu | 70.94 |
| Kuwait | 28.08 | Venezuela, RB | 18.23 |
| Kyrgyz Republic | 32.51 | Vietnam | 9.85 |
| Lao PDR | 4.43 | Yemen, Rep. | 5.91 |
| Latvia | 74.38 | Zambia | 35.47 |
| Lebanon | 31.53 | Zimbabwe | 19.70 |
| Lesotho | 47.78 | China | 6.90 |
| Liberia | 43.35 |  |  |

**Table 2: 2^nd^ indicator: Political stability**

| Country/Territory | Percentile rank | Country/Territory | Percentile rank |
| --- | --- | --- | --- |
| Afghanistan | 0.95 | Libya | 3.81 |
| Albania | 55.24 | Liechtenstein | 98.57 |
| Algeria | 11.43 | Lithuania | 71.43 |
| Andorra | 97.14 | Luxembourg | 97.62 |
| Angola | 31.90 | Macedonia, FYR | 32.38 |
| Antigua and Barbuda | 87.62 | Madagascar | 31.43 |
| Argentina | 53.81 | Malawi | 45.24 |
| Armenia | 24.76 | Malaysia | 50.00 |
| Australia | 81.90 | Maldives | 60.00 |
| Austria | 72.86 | Mali | 8.57 |
| Azerbaijan | 17.62 | Malta | 89.52 |
| Bahamas, The | 78.10 | Marshall Islands | 80.48 |
| Bahrain | 18.10 | Mauritania | 20.95 |
| Bangladesh | 10.48 | Mauritius | 88.57 |
| Barbados | 81.43 | Mexico | 20.00 |
| Belarus | 50.48 | Micronesia, Fed. Sts. | 89.05 |
| Belgium | 61.43 | Moldova | 36.19 |
| Belize | 49.05 | Monaco | 85.71 |
| Benin | 48.57 | Mongolia | 73.33 |
| Bhutan | 82.86 | Montenegro | 50.95 |
| Bolivia | 37.62 | Morocco | 35.71 |
| Bosnia and Herzegovina | 32.86 | Mozambique | 12.38 |
| Botswana | 90.00 | Myanmar | 23.33 |
| Brazil | 30.00 | Namibia | 70.00 |
| Brunei Darussalam | 93.81 | Nauru | 64.76 |
| Bulgaria | 47.14 | Nepal | 19.05 |
| Burkina Faso | 15.24 | Netherlands | 77.62 |
| Burundi | 5.24 | New Zealand | 99.05 |
| Cambodia | 52.38 | Nicaragua | 39.52 |
| Cameroon | 14.76 | Niger | 11.90 |
| Canada | 93.33 | Nigeria | 6.67 |
| Cape Verde | 77.14 | Norway | 91.43 |
| Central African Republic | 7.14 | Oman | 71.90 |
| Chad | 10.95 | Pakistan | 1.43 |
| Chile | 63.81 | Palau | 80.48 |
| Colombia | 13.81 | Panama | 60.48 |
| Comoros | 46.67 | Papua New Guinea | 29.05 |
| Congo, Rep. | 25.24 | Paraguay | 53.33 |
| Congo, Dem. Rep. | 4.29 | Peru | 40.95 |
| Costa Rica | 70.48 | Philippines | 10.00 |
| Côte d'Ivoire | 16.19 | Poland | 63.33 |
| Croatia | 68.10 | Portugal | 88.10 |
| Cuba | 66.19 | Qatar | 76.19 |
| Cyprus | 65.71 | Romania | 55.71 |
| Czech Republic | 83.33 | Russian Federation | 16.67 |
| Denmark | 74.76 | Rwanda | 45.71 |
| Djibouti | 23.81 | St. Kitts and Nevis | 67.14 |
| Dominica | 90.95 | St. Lucia | 75.24 |
| Dominican Republic | 57.14 | St. Vincent and the Grenadines | 87.62 |
| Ecuador | 42.86 | Samoa | 92.38 |
| Timor-Leste | 43.33 | San Marino | 80.48 |
| Egypt, Arab Rep. | 9.05 | São Tomé and Principe | 54.29 |
| El Salvador | 44.76 | Saudi Arabia | 28.57 |
| Equatorial Guinea | 39.05 | Senegal | 36.67 |
| Eritrea | 17.14 | Serbia | 48.10 |
| Estonia | 68.57 | Seychelles | 69.52 |
| Ethiopia | 7.62 | Sierra Leone | 40.48 |
| Fiji | 73.81 | Singapore | 99.52 |
| Finland | 80.95 | Slovak Republic | 66.67 |
| France | 44.29 | Slovenia | 83.81 |
| Gabon | 43.81 | Solomon Islands | 62.86 |
| Gambia, The | 27.62 | Somalia | 2.86 |
| Georgia | 35.24 | South Africa | 42.38 |
| Germany | 70.95 | Spain | 61.90 |
| Ghana | 40.00 | Sri Lanka | 49.52 |
| Greece | 41.90 | Sudan | 2.38 |
| Grenada | 87.62 | South Sudan | 1.90 |
| Guatemala | 26.19 | Suriname | 56.19 |
| Guinea | 30.95 | Swaziland | 29.52 |
| Guinea-Bissau | 28.10 | Sweden | 82.38 |
| Guyana | 46.19 | Switzerland | 95.71 |
| Haiti | 22.38 | Syrian Arab Republic | 0.00 |
| Honduras | 33.81 | Tajikistan | 19.52 |
| Hungary | 69.05 | Tanzania | 30.48 |
| Iceland | 96.19 | Thailand | 15.71 |
| India | 14.29 | Togo | 38.57 |
| Indonesia | 33.33 | Tonga | 78.57 |
| Iran, Islamic Rep. | 20.48 | Trinidad and Tobago | 56.67 |
| Iraq | 3.33 | Tunisia | 13.33 |
| Ireland | 76.67 | Turkey | 5.71 |
| Israel | 18.57 | Turkmenistan | 34.76 |
| Italy | 58.10 | Tuvalu | 97.14 |
| Jamaica | 54.76 | Uganda | 21.43 |
| Japan | 86.19 | Ukraine | 6.19 |
| Jordan | 26.67 | United Arab Emirates | 60.95 |
| Kazakhstan | 47.62 | United Kingdom | 59.05 |
| Kenya | 9.52 | United States | 58.57 |
| Kiribati | 75.71 | Uruguay | 90.48 |
| Korea, Rep. | 51.90 | Uzbekistan | 34.29 |
| Korea, Dem. Rep. | 21.90 | Vanuatu | 64.29 |
| Kuwait | 41.43 | Venezuela, RB | 12.86 |
| Kyrgyz Republic | 22.86 | Vietnam | 51.43 |
| Lao PDR | 62.38 | Yemen, Rep. | 0.48 |
| Latvia | 59.52 | Zambia | 52.86 |
| Lebanon | 8.10 | Zimbabwe | 24.29 |
| Lesotho | 37.14 | China | 27.14 |
| Liberia | 25.71 |  |  |

**Table 3: 6^th^ indicator: Control of corruption**

| Country/Territory | Percentile rank | Country/Territory | Percentile rank |
| --- | --- | --- | --- |
| Afghanistan | 3.37 | Libya | 2.88 |
| Albania | 41.35 | Liechtenstein | 96.63 |
| Algeria | 27.40 | Lithuania | 73.08 |
| Andorra | 87.50 | Luxembourg | 97.60 |
| Angola | 5.77 | Macedonia, FYR | 46.63 |
| Antigua and Barbuda | 75.00 | Madagascar | 16.35 |
| Argentina | 46.15 | Malawi | 24.04 |
| Armenia | 32.69 | Malaysia | 61.54 |
| Australia | 93.27 | Maldives | 28.85 |
| Austria | 91.35 | Mali | 29.81 |
| Azerbaijan | 17.79 | Malta | 75.96 |
| Bahamas, The | 82.69 | Marshall Islands | 55.77 |
| Bahrain | 56.25 | Mauritania | 21.63 |
| Bangladesh | 21.15 | Mauritius | 65.38 |
| Barbados | 87.98 | Mexico | 23.08 |
| Belarus | 47.60 | Micronesia, Fed. Sts. | 72.12 |
| Belgium | 92.31 | Moldova | 14.42 |
| Belize | 49.52 | Mongolia | 35.58 |
| Benin | 36.54 | Montenegro | 54.33 |
| Bhutan | 83.17 | Morocco | 52.88 |
| Bolivia | 26.92 | Mozambique | 18.27 |
| Bosnia and Herzegovina | 37.02 | Myanmar | 30.77 |
| Botswana | 80.29 | Namibia | 65.87 |
| Brazil | 38.46 | Nauru | 37.98 |
| Brunei Darussalam | 72.60 | Nepal | 23.56 |
| Bulgaria | 51.44 | Netherlands | 94.71 |
| Burkina Faso | 53.37 | New Zealand | 100.00 |
| Burundi | 10.58 | Nicaragua | 17.31 |
| Cambodia | 8.17 | Niger | 31.25 |
| Cameroon | 11.06 | Nigeria | 13.46 |
| Canada | 95.19 | Norway | 98.08 |
| Cape Verde | 79.33 | Oman | 66.35 |
| Central African Republic | 9.13 | Pakistan | 19.23 |
| Chad | 4.81 | Palau | 37.98 |
| Chile | 82.21 | Panama | 36.06 |
| Colombia | 44.23 | Papua New Guinea | 15.87 |
| Comoros | 31.73 | Paraguay | 25.00 |
| Congo, Rep. | 9.62 | Peru | 43.27 |
| Congo, Dem. Rep. | 7.69 | Philippines | 34.13 |
| Costa Rica | 75.48 | Poland | 76.44 |
| Côte d'Ivoire | 33.65 | Portugal | 80.77 |
| Croatia | 62.50 | Qatar | 79.81 |
| Cuba | 60.58 | Romania | 58.17 |
| Cyprus | 77.88 | Russian Federation | 18.75 |
| Czech Republic | 67.79 | Rwanda | 74.52 |
| Denmark | 99.04 | St. Kitts and Nevis | 68.27 |
| Djibouti | 30.29 | St. Lucia | 70.67 |
| Dominica | 70.19 | St. Vincent and the Grenadines | 74.04 |
| Dominican Republic | 22.60 | Samoa | 64.90 |
| Ecuador | 29.33 | São Tomé and Principe | 55.29 |
| Timor-Leste | 34.62 | Saudi Arabia | 62.98 |
| Egypt, Arab Rep. | 32.21 | Senegal | 57.21 |
| El Salvador | 33.17 | Serbia | 45.67 |
| Equatorial Guinea | 0.00 | Seychelles | 76.92 |
| Eritrea | 11.54 | Sierra Leone | 20.19 |
| Estonia | 84.62 | Singapore | 97.12 |
| Ethiopia | 39.90 | Slovak Republic | 63.46 |
| Fiji | 62.02 | Slovenia | 77.40 |
| Finland | 99.52 | Solomon Islands | 43.75 |
| France | 90.38 | Somalia | 0.48 |
| Gabon | 24.52 | South Africa | 60.10 |
| Gambia, The | 22.12 | Spain | 68.75 |
| Georgia | 73.56 | Sri Lanka | 48.08 |
| Germany | 93.75 | Sudan | 1.44 |
| Ghana | 50.96 | South Sudan | 1.92 |
| Greece | 56.73 | Suriname | 44.71 |
| Grenada | 69.71 | Swaziland | 39.42 |
| Guatemala | 25.48 | Sweden | 98.56 |
| Guinea | 14.90 | Switzerland | 96.15 |
| Guinea-Bissau | 3.85 | Syrian Arab Republic | 2.40 |
| Guyana | 45.19 | Tajikistan | 12.50 |
| Haiti | 7.21 | Tanzania | 35.10 |
| Honduras | 27.88 | Thailand | 40.87 |
| Hungary | 61.06 | Togo | 28.37 |
| Iceland | 95.67 | Tonga | 38.94 |
| India | 47.12 | Trinidad and Tobago | 48.56 |
| Indonesia | 42.79 | Tunisia | 53.85 |
| Iran, Islamic Rep. | 25.96 | Turkey | 50.48 |
| Iraq | 6.25 | Turkmenistan | 4.33 |
| Ireland | 92.79 | Tuvalu | 58.65 |
| Israel | 81.73 | Uganda | 12.98 |
| Italy | 59.62 | Ukraine | 19.71 |
| Jamaica | 51.92 | United Arab Emirates | 88.46 |
| Japan | 90.87 | United Kingdom | 94.23 |
| Jordan | 64.42 | United States | 89.90 |
| Kazakhstan | 20.67 | Uruguay | 89.42 |
| Kenya | 16.83 | Uzbekistan | 10.10 |
| Kiribati | 63.94 | Vanuatu | 54.81 |
| Korea, Rep. | 66.83 | Venezuela, RB | 6.73 |
| Korea, Dem. Rep. | 5.29 | Vietnam | 41.83 |
| Kuwait | 50.00 | Yemen, Rep. | 0.96 |
| Kyrgyz Republic | 12.02 | Zambia | 42.31 |
| Lao PDR | 15.38 | Zimbabwe | 8.65 |
| Latvia | 67.31 | China | 49.04 |
| Lebanon | 13.94 |  |  |
| Lesotho | 57.69 |  |  |
| Liberia | 26.44 |  |  |

1. Factors affecting women

**Table 4: Gross National Income for females (GNI- F)**

| Country/ territory | GNI of females | Country/ territory | GNI of females |
| --- | --- | --- | --- |
| Angola | 5073 | Mexico | 10710 |
| Bahamas | 18070 | Mongolia | 8809 |
| Cape Verde | 4030 | Montenegro | 11757 |
| Djibouti | 1981 | New Zealand | 24413 |
| Equatorial Guinea | 17462 | Nicaragua | 3150 |
| Eritrea | 1286 | Paraguay | 6138 |
| Fiji | 4695 | Peru | 8939 |
| Guinea-Bissau | 1139 | Serbia | 9600 |
| Haiti | 1370 | South Africa | 8795 |
| Malaysia | 17170 | Spain | 24382 |
| Myanmar | 4182 | Suriname | 10501 |
| Papua New Guinea | 2362 | Switzerland | 46798 |
| St. Vincent & the Grenadines | 7600 | Tonga | 3959 |
| Samoa | 3444 | United Arab Emirates | 27257 |
| Solomon Is. | 1061 | United Kingdom | 26324 |
| Somalia | 170 | Venezuela | 11579 |
| Turkmenistan | 9359 | China | 10705 |
| Vanuatu | 2139 | Azerbaijan | 11029 |
| Argentina | 12875 | Bangladesh | 2379 |
| Armenia | 5535 | Bolivia | 4695 |
| Australia | 34271 | Congo | 4731 |
| Barbados | 11801 | Cuba | 5013 |
| Belarus | 12327 | Guyana | 4346 |
| Belgium | 32416 | Honduras | 2680 |
| Botswana | 13278 | Indonesia | 6668 |
| Brazil | 10672 | South Korea | 21308 |
| Brunei | 55402 | Macedonia | 9050 |
| Bulgaria | 12979 | Madagascar | 1091 |
| Canada | 33288 | Maldives | 7155 |
| Colombia | 10215 | Netherlands | 30117 |
| Croatia | 16932 | Oman | 15703 |
| Cyprus | 23450 | Sri Lanka | 6067 |
| Czech Republic | 20997 | Tajikistan | 2100 |
| Dominican Republic | 9281 | Tanzania | 2359 |
| Ecuador | 8278 | Uzbekistan | 3891 |
| Finland | 32069 | Zimbabwe | 1360 |
| France | 31742 | Bosnia & Herzegovina | 6950 |
| Hungary | 17787 | Burundi | 632 |
| Ireland | 33497 | Gabon | 15838 |
| Kazakhstan | 16364 | Kenya | 2357 |
| Moldova | 4461 | Laos | 4408 |
| Namibia | 7971 | Malawi | 972 |
| Norway | 59800 | Malta | 17295 |
| Panama | 14550 | Nepal | 1979 |
| Philippines | 6845 | Sao Tome & Principe | 2000 |
| Poland | 18928 | Tunisia | 4662 |
| Portugal | 21095 | Turkey | 10648 |
| Qatar | 50324 | Zambia | 2803 |
| Romania | 16272 | Afghanistan | 511 |
| Russia | 17868 | Algeria | 4022 |
| Rwanda | 1428 | Benin | 1673 |
| St. Lucia | 8033 | Bhutan | 5657 |
| Singapore | 60787 | Burkina Faso | 1278 |
| Slovakia | 20173 | Cambodia | 2650 |
| Slovenia | 25654 | Cameroon | 2340 |
| Sweden | 40328 | Central African Republic | 482 |
| Thailand | 12938 | Chad | 1581 |
| Trinidad & Tobago | 21104 | Comoros | 715 |
| Ukraine | 5791 | Congo, DRC | 599 |
| United States | 42272 | Cote d'Ivoire | 2136 |
| Uruguay | 14608 | East Timor | 3124 |
| Vietnam | 4834 | Egypt | 4750 |
| Albania | 7365 | Ethiopia | 1161 |
| Austria | 29829 | Gambia | 1296 |
| Bahrain | 25717 | Ghana | 3200 |
| Belize | 5360 | Guinea | 848 |
| Chile | 14955 | India | 2184 |
| Costa Rica | 9955 | Iran | 5132 |
| Denmark | 36857 | Iraq | 3552 |
| El Salvador | 5386 | Jordan | 3203 |
| Estonia | 21976 | Lebanon | 5844 |
| Georgia | 6105 | Liberia | 575 |
| Germany | 35878 | Mali | 1349 |
| Greece | 17304 | Mauritania | 1608 |
| Guatemala | 5132 | Morocco | 3388 |
| Iceland | 30530 | Mozambique | 1016 |
| Israel | 23323 | Niger | 481 |
| Italy | 22910 | Nigeria | 4132 |
| Jamaica | 6628 | Pakistan | 1498 |
| Japan | 25385 | Saudi Arabia | 19300 |
| Kuwait | 35164 | Senegal | 1706 |
| Kyrgyzstan | 2123 | Sierra Leone | 1354 |
| Latvia | 18824 | North Sudan | 1902 |
| Lesotho | 2631 | Swaziland | 5078 |
| Libya | 7163 | Syria | 835 |
| Lithuania | 22147 | Togo | 1116 |
| Luxembourg | 47539 | Uganda | 1266 |
| Mauritius | 10540 | Yemen | 1045 |

**Table 5: Female share of parliament seats (%)**

| Country | % | Country | % |
| --- | --- | --- | --- |
| Afghanistan | 27.4 | Libya | 16.0 |
| Albania | 20.7 | Liechtenstein | 20.0 |
| Algeria | 25.7 | Lithuania | 23.4 |
| Andorra | 39.3 | Luxembourg | 28.3 |
| Angola | 36.8 | The former Yugoslav Republic of Macedonia | 33.3 |
| Antigua and Barbuda | 25.7 | Madagascar | 20.5 |
| Argentina | 37.1 | Malawi | 16.7 |
| Armenia | 10.7 | Malaysia | 13.2 |
| Australia | 30.5 | Maldives | 5.9 |
| Austria | 30.3 | Mali | 8.8 |
| Azerbaijan | 16.9 | Malta | 12.9 |
| Bahamas | 16.7 | Marshall Islands | 9.1 |
| Bahrain | 15.0 | Mauritania | 22.2 |
| Bangladesh | 20.0 | Mauritius | 11.6 |
| Barbados | 19.6 | Mexico | 40.6 |
| Belarus | 29.2 | Micronesia (Federated States of) | 0.0 |
| Belgium | 42.4 | Moldova (Republic of) | 21.8 |
| Belize | 13.3 | Monaco | 20.8 |
| Benin | 7.2 | Mongolia | 14.5 |
| Bhutan | 8.3 | Montenegro | 17.3 |
| Bolivia (Plurinational State of) | 51.8 | Morocco | 15.7 |
| Bosnia and Herzegovina | 19.3 | Mozambique | 39.6 |
| Botswana | 9.5 | Myanmar | 13.0 |
| Brazil | 10.8 | Namibia | 37.7 |
| Bulgaria | 20.4 | Nauru | 5.3 |
| Burkina Faso | 9.4 | Nepal | 29.5 |
| Burundi | 37.8 | Netherlands | 36.4 |
| Cambodia | 19.0 | New Zealand | 31.4 |
| Cameroon | 27.1 | Nicaragua | 41.3 |
| Canada | 28.3 | Niger | 13.3 |
| Cabo Verde | 20.8 | Nigeria | 5.8 |
| Central African Republic | 12.5 | Norway | 39.6 |
| Chad | 14.9 | Oman | 8.2 |
| Chile | 15.8 | Pakistan | 20.0 |
| Colombia | 20.9 | Palau | 10.3 |
| Comoros | 3.0 | Panama | 18.3 |
| Congo | 11.5 | Papua New Guinea | 2.7 |
| Congo (Democratic Republic of the) | 8.2 | Paraguay | 16.8 |
| Costa Rica | 33.3 | Peru | 22.3 |
| Côte d'Ivoire | 9.2 | Philippines | 27.1 |
| Croatia | 15.2 | Poland | 24.8 |
| Cuba | 48.9 | Portugal | 34.8 |
| Cyprus | 12.5 | Qatar | 0.0 |
| Czech Republic | 19.6 | Romania | 12.0 |
| Denmark | 37.4 | Russian Federation | 14.5 |
| Djibouti | 12.7 | Rwanda | 57.5 |
| Dominica | 21.9 | Saint Kitts and Nevis | 13.3 |
| Dominican Republic | 19.1 | Saint Lucia | 20.7 |
| Ecuador | 41.6 | Saint Vincent and the Grenadines | 13.0 |
| Timor-Leste | 38.5 | Samoa | 6.1 |
| Egypt | 2.2 | San Marino | 16.7 |
| El Salvador | 32.1 | Sao Tome and Principe | 18.2 |
| Equatorial Guinea | 19.7 | Saudi Arabia | 19.9 |
| Eritrea | 22.0 | Senegal | 42.7 |
| Estonia | 23.8 | Serbia | 34.0 |
| Ethiopia | 37.3 | Seychelles | 43.8 |
| Fiji | 16.0 | Sierra Leone | 12.4 |
| Finland | 41.5 | Singapore | 23.9 |
| France | 25.7 | Slovakia | 18.7 |
| Gabon | 16.0 | Slovenia | 27.7 |
| Gambia | 9.4 | Solomon Islands | 2.0 |
| Georgia | 11.3 | Somalia | 13.8 |
| Germany | 36.9 | South Africa | 41.2 |
| Ghana | 10.9 | Spain | 38.0 |
| Greece | 19.7 | Sri Lanka | 4.9 |
| Grenada | 25.0 | Sudan | 31.0 |
| Guatemala | 13.9 | South Sudan | 24.3 |
| Guinea | 21.9 | Suriname | 25.5 |
| Guinea-Bissau | 13.7 | Swaziland | 14.7 |
| Guyana | 30.4 | Sweden | 43.6 |
| Haiti | 3.5 | Switzerland | 28.9 |
| Honduras | 25.8 | Syrian Arab Republic | 12.4 |
| Hungary | 10.1 | Tajikistan | 14.7 |
| Iceland | 41.3 | Tanzania (United Republic of) | 36.0 |
| India | 12.2 | Thailand | 6.1 |
| Indonesia | 17.1 | Togo | 17.6 |
| Iran (Islamic Republic of) | 3.1 | Tonga | 0.0 |
| Iraq | 26.5 | Trinidad and Tobago | 31.5 |
| Ireland | 19.9 | Tunisia | 31.3 |
| Israel | 26.7 | Turkey | 14.9 |
| Italy | 30.1 | Turkmenistan | 25.8 |
| Jamaica | 16.7 | Tuvalu | 6.7 |
| Japan | 11.6 | Uganda | 35.0 |
| Jordan | 11.6 | Ukraine | 12.1 |
| Kazakhstan | 20.1 | United Arab Emirates | 22.5 |
| Kenya | 20.8 | United Kingdom | 26.7 |
| Kiribati | 8.7 | United States | 19.5 |
| Korea (Republic of) | 16.3 | Uruguay | 19.2 |
| Kuwait | 1.5 | Uzbekistan | 16.4 |
| Kyrgyzstan | 19.2 | Vanuatu | 0.0 |
| Lao People's Democratic Republic | 25.0 | Venezuela (Bolivarian Republic of) | 17.0 |
| Latvia | 18.0 | Viet Nam | 24.3 |
| Lebanon | 3.1 | Yemen | 0.5 |
| Lesotho | 24.8 | Zambia | 12.7 |
| Liberia | 10.7 | Zimbabwe | 35.1 |

**Table 6: Legislators, senior officials and managers, female (% of total)**

| Country | % | Country | % |
| --- | --- | --- | --- |
| Algeria | 10.6 | Luxembourg | 24.45 |
| Austria | 28.05 | Macedonia | 27.85 |
| Bahamas | 51.6 | Madagascar | 25.1 |
| Bangladesh | 5.4 | Malaysia | 25 |
| Barbados | 47.03333 | Malta | 23 |
| Belgium | 32 | Moldova | 39.93333 |
| Bhutan | 30.125 | Montenegro | 30.43333 |
| Botswana | 38.6 | Namibia | 38.66667 |
| Brazil | 37.06667 | Netherlands | 29.1 |
| Brunei | 33.7 | Norway | 32.95 |
| Bulgaria | 35.45 | Panama | 46.95 |
| Canada | 36.45 | Paraguay | 34.14 |
| Colombia | 53.1 | Peru | 30.9 |
| Costa Rica | 32.45 | Poland | 37 |
| Croatia | 25.3 | Portugal | 32.5 |
| Cyprus | 13.7 | Qatar | 12.2 |
| Czech Republic | 26.75 | Romania | 31.15 |
| Denmark | 25.25 | Russia | 38.925 |
| Dominica | 38.025 | Samoa | 36.3 |
| Ecuador | 40.2 | Sao Tome & Principe | 24.4 |
| East Timor | 10.3 | Saudi Arabia | 6.8 |
| Egypt | 11.05 | Serbia | 32.7 |
| El Salvador | 33.05 | Singapore | 34.1 |
| Estonia | 36.15 | Slovakia | 32.8 |
| Ethiopia | 21 | Slovenia | 36.65 |
| Finland | 31.2 | South Africa | 30.66667 |
| France | 38.95 | Spain | 32.05 |
| Germany | 30.1 | Sri Lanka | 26.9 |
| Greece | 26.6 | Suriname | 35.8 |
| Hungary | 38.45 | Sweden | 33 |
| Iceland | 37.15 | Switzerland | 33 |
| India | 13.8 | Syria | 9.2 |
| Indonesia | 22.2 | Thailand | 25.45 |
| Ireland | 36.2 | Turkey | 10 |
| Italy | 28.9 | Ukraine | 39.5 |
| Kiribati | 36.5 | United Kingdom | 35 |
| Latvia | 43.55 | United States | 43.275 |
| Lithuania | 39.8 | Uruguay | 44.45 |
|  |  | Yemen | 5.2 |

**Table 7: Youth unemployment rate, female to male ratio**

| Country | Ratio | Country | Ratio |
| --- | --- | --- | --- |
| Afghanistan | 1.01 | Lebanon | 1.07 |
| Albania | 0.93 | Lesotho | 1.21 |
| Algeria | 1.15 | Liberia | 1.22 |
| Angola | 1.08 | Libya | 1.2 |
| Argentina | 1.13 | Lithuania | 1.02 |
| Armenia | 1.15 | Luxembourg | 0.85 |
| Australia | 0.94 | Macedonia | 0.96 |
| Austria | 0.96 | Madagascar | 1.13 |
| Azerbaijan | 1.11 | Malawi | 0.95 |
| Bahamas | 1.16 | Malaysia | 1.09 |
| Bahrain | 2.01 | Maldives | 0.93 |
| Bangladesh | 1.03 | Mali | 1.35 |
| Barbados | 1.09 | Malta | 0.85 |
| Belarus | 1.03 | Mauritania | 1.19 |
| Belgium | 0.96 | Mauritius | 1.21 |
| Belize | 1.55 | Mexico | 1.06 |
| Benin | 1.56 | Moldova | 0.83 |
| Bhutan | 1.08 | Mongolia | 1.06 |
| Bolivia | 1.2 | Montenegro | 1 |
| Bosnia & Herzegovina | 1.01 | Morocco | 0.98 |
| Botswana | 1.16 | Mozambique | 0.98 |
| Brazil | 1.26 | Myanmar | 1.1 |
| Brunei | 1.09 | Namibia | 1.11 |
| Bulgaria | 1.03 | Nepal | 0.81 |
| Burkina Faso | 0.83 | Netherlands | 1.07 |
| Burundi | 1.07 | New Zealand | 1.02 |
| Cambodia | 0.79 | Nicaragua | 1.14 |
| Cameroon | 1.2 | Niger | 0.8 |
| Canada | 0.88 | Nigeria | 1.27 |
| Cape Verde | 1.33 | Norway | 0.86 |
| Central African Republic | 1.06 | Oman | 1.22 |
| Chad | 1.21 | Pakistan | 1.12 |
| Chile | 1.19 | Panama | 1.14 |
| Colombia | 1.28 | Papua New Guinea | 1.07 |
| Comoros | 1.02 | Paraguay | 1.27 |
| Congo | 1.08 | Peru | 1.04 |
| Congo, DRC | 1.23 | Philippines | 1.07 |
| Costa Rica | 1.15 | Poland | 1.02 |
| Cote d'Ivoire | 1.24 | Portugal | 1.12 |
| Croatia | 0.99 | Qatar | 2.13 |
| Cuba | 1.03 | Romania | 1.02 |
| Cyprus | 0.91 | Russia | 1.05 |
| Czech Republic | 1.09 | Rwanda | 1.07 |
| Denmark | 0.95 | St. Lucia | 1.39 |
| Dominican Republic | 1.43 | Samoa | 1.14 |
| Ecuador | 1.31 | Sao Tome & Principe | 1.13 |
| East Timor | 1.28 | Saudi Arabia | 1.36 |
| Egypt | 1.3 | Senegal | 1.35 |
| El Salvador | 0.98 | Serbia | 1.04 |
| Equatorial Guinea | 1.1 | Sierra Leone | 0.7 |
| Eritrea | 1.09 | Singapore | 1.32 |
| Estonia | 0.95 | Slovakia | 0.98 |
| Ethiopia | 1.61 | Slovenia | 0.9 |
| Fiji | 1.23 | Solomon Is. | 1 |
| Finland | 0.87 | Somalia | 1.06 |
| France | 0.98 | South Africa | 1.07 |
| Gabon | 1.16 | Spain | 0.99 |
| Gambia | 1.15 | Sri Lanka | 1.32 |
| Georgia | 1.04 | North Sudan | 1.2 |
| Germany | 0.92 | Suriname | 1.61 |
| Ghana | 1.12 | Swaziland | 1.02 |
| Greece | 1.11 | Sweden | 0.94 |
| Guatemala | 1.2 | Switzerland | 0.96 |
| Guinea | 0.69 | Syria | 1.25 |
| Guinea-Bissau | 1.08 | Tajikistan | 0.9 |
| Guyana | 1.22 | Tanzania | 1.22 |
| Haiti | 1.18 | Thailand | 1.12 |
| Honduras | 1.3 | Togo | 1.09 |
| Hungary | 0.95 | Tonga | 1.2 |
| Iceland | 0.82 | Trinidad & Tobago | 1.26 |
| India | 1.01 | Tunisia | 1.02 |
| Indonesia | 1.06 | Turkey | 1.13 |
| Iran | 1.17 | Turkmenistan | 1.04 |
| Iraq | 1.14 | Uganda | 1.04 |
| Ireland | 0.9 | Ukraine | 0.95 |
| Israel | 1.05 | United Arab Emirates | 1.21 |
| Italy | 1.07 | United Kingdom | 0.9 |
| Jamaica | 1.28 | United States | 0.92 |
| Japan | 0.99 | Uruguay | 1.1 |
| Jordan | 1.19 | Uzbekistan | 1.05 |
| Kazakhstan | 1.16 | Vanuatu | 1.06 |
| Kenya | 1.03 | Venezuela | 1.16 |
| South Korea | 0.92 | Vietnam | 1.1 |
| North Korea | 1.01 | Yemen | 1.17 |
| Kuwait | 0.91 | Zambia | 0.96 |
| Kyrgyzstan | 1.14 | Zimbabwe | 1.12 |
| Laos | 0.78 | China | 0.94 |
| Latvia | 1.01 |  |  |

**Table 8: Mandatory paid maternity leave (days)**

| Country | Days | Country | Days |
| --- | --- | --- | --- |
| Afghanistan | 90 | Kyrgyzstan | 126 |
| Albania | 365 | Laos | 105 |
| Algeria | 98 | Latvia | 112 |
| Angola | 90 | Lebanon | 70 |
| Antigua & Barbuda | 91 | Lesotho | 84 |
| Argentina | 90 | Liberia | 90 |
| Armenia | 140 | Libya | 98 |
| Austria | 112 | Lithuania | 126 |
| Azerbaijan | 126 | Luxembourg | 112 |
| Bahamas | 91 | Macedonia | 270 |
| Bahrain | 60 | Madagascar | 98 |
| Bangladesh | 112 | Malawi | 56 |
| Barbados | 84 | Malaysia | 60 |
| Belarus | 126 | Maldives | 60 |
| Belgium | 105 | Mali | 98 |
| Belize | 98 | Malta | 126 |
| Benin | 98 | Mauritania | 98 |
| Bhutan | 56 | Mauritius | 84 |
| Bolivia | 90 | Mexico | 84 |
| Bosnia & Herzegovina | 365 | Moldova | 126 |
| Botswana | 84 | Mongolia | 120 |
| Brazil | 120 | Montenegro | 45 |
| Brunei | 91 | Morocco | 98 |
| Bulgaria | 410 | Mozambique | 60 |
| Burkina Faso | 98 | Myanmar | 98 |
| Burundi | 84 | Namibia | 84 |
| Cambodia | 90 | Nepal | 52 |
| Cameroon | 98 | Netherlands | 112 |
| Canada | 105 | New Zealand | 112 |
| Cape Verde | 60 | Nicaragua | 84 |
| Central African Republic | 98 | Niger | 98 |
| Chad | 98 | Nigeria | 84 |
| Chile | 126 | Oman | 50 |
| Colombia | 98 | Pakistan | 84 |
| Comoros | 98 | Panama | 98 |
| Congo | 105 | Papua New Guinea | 0 |
| Congo, DRC | 98 | Paraguay | 63 |
| Costa Rica | 120 | Peru | 90 |
| Cote d'Ivoire | 98 | Philippines | 60 |
| Croatia | 208 | Poland | 182 |
| Cyprus | 126 | Qatar | 50 |
| Czech Republic | 196 | Romania | 126 |
| Denmark | 126 | Russia | 140 |
| Djibouti | 98 | Rwanda | 84 |
| Dominica | 84 | St. Kitts & Nevis | 91 |
| Dominican Republic | 84 | St. Lucia | 91 |
| Ecuador | 84 | Samoa | 28 |
| East Timor | 84 | San Marino | 150 |
| Egypt | 90 | Sao Tome & Principe | 90 |
| El Salvador | 84 | Saudi Arabia | 70 |
| Equatorial Guinea | 84 | Senegal | 98 |
| Eritrea | 60 | Serbia | 135 |
| Estonia | 140 | Seychelles | 98 |
| Ethiopia | 90 | Sierra Leone | 84 |
| Fiji | 84 | Singapore | 105 |
| Finland | 147 | Slovakia | 238 |
| France | 112 | Slovenia | 105 |
| Gabon | 98 | Solomon Is. | 84 |
| Gambia | 180 | South Africa | 120 |
| Georgia | 183 | Spain | 112 |
| Germany | 98 | Sri Lanka | 84 |
| Ghana | 84 | North Sudan | 56 |
| Greece | 119 | South Sudan | 56 |
| Grenada | 90 | Suriname | 0 |
| Guatemala | 84 | Swaziland | 14 |
| Guinea | 98 | Switzerland | 98 |
| Guinea-Bissau | 60 | Syria | 120 |
| Guyana | 91 | Tajikistan | 140 |
| Haiti | 42 | Tanzania | 84 |
| Honduras | 84 | Thailand | 90 |
| Hungary | 168 | Togo | 98 |
| Iceland | 90 | Tonga | 0 |
| India | 84 | Trinidad & Tobago | 98 |
| Indonesia | 90 | Tunisia | 30 |
| Iran | 270 | Turkey | 112 |
| Iraq | 72 | Uganda | 84 |
| Ireland | 182 | Ukraine | 126 |
| Israel | 98 | United Arab Emirates | 45 |
| Italy | 150 | United Kingdom | 14 |
| Jamaica | 56 | Uruguay | 98 |
| Japan | 98 | Uzbekistan | 126 |
| Jordan | 70 | Vanuatu | 84 |
| Kazakhstan | 126 | Venezuela | 182 |
| Kenya | 90 | Vietnam | 180 |
| Kiribati | 84 | Yemen | 70 |
| South Korea | 90 | Zambia | 84 |
| Kuwait | 70 | Zimbabwe | 98 |
|  |  | China | 128 |

Table 9: Comparison between countries with and without missing ECC data

| Variables | Countries with ECC data | Countries without ECC data | P value |
| --- | --- | --- | --- |
| GNI per capita- females | 16,505.6 (14,735.0) | 7,367.2 (8,217.1) | <0.0001* |
| Females’ share of parliament seats, % of total | 22.3 (11.4) | 19.4 (11.5) | 0.08 |
| Legislators, senior officials and managers, females % of total | 30.3 (9.6) | 30.7 (12.0) | 0.88 |
| Youth unemployment rate, female: male ratio | 1.1 (0.2) | 1.1 (0.2) | 0.30 |
| Mandatory paid maternity leave in days | 112.8 (55.9) | 95.1 (51.7) | 0.03* |
| Voice and accountability, rank | 51.8 (29.9) | 46.9 (28.3) | 0.24 |
| Political stability and absence of violence/ terrorism, rank | 45.6 (27.7) | 49.1 (28.8) | 0.39 |
| Control of corruption, rank | 52.7 (30.2) | 43.2 (27.2) | 0.03* |

*: statistically significant at P< 0.05. Differences between groups in all variables were assessed using t test except for GNI per capita- females where Mann Whitney U test was used
